# Biomechanical Comparison Between Porous Ti6Al4V Block and Tumor Prosthesis UHMWPE Block for the Treatment of Distal Femur Bone Defects

**DOI:** 10.3389/fbioe.2022.939371

**Published:** 2022-07-05

**Authors:** Jiangbo Zhang, Yang Liu, Qing Han, Aobo Zhang, Hao Chen, Mingyue Ma, Yongyue Li, Bingpeng Chen, Jincheng Wang

**Affiliations:** ^1^ Department of Orthopedics, The Second Hospital of Jilin University, Changchun, China; ^2^ Department of Breast Surgery, The First Hospital of Jilin University, Changchun, China

**Keywords:** finite element analysis, total knee revision, bone defect, distal femur, UHMWPE block, metal block

## Abstract

**Purpose:** The management of bone defects is a crucial content of total knee revision. This study compared the biomechanical performance of porous Ti6Al4V block and tumor prosthesis UHMWPE block in treating distal femoral bone defects.

**Methods:** The finite element models of AORI type 3 distal femoral bone defect treated with porous Ti6Al4V block and UHMWPE block were established. Sensitivity analysis was performed to obtain the appropriate mesh size. The biomechanical performance of treatment methods in bone defects were evaluated according to the peak stress, the Von Mises stress distribution, and the average stresses of regions of interest under the condition of standing on one foot and flexion of the knee. Statistical analysis was conducted by independent samples *t*-test in SPSS (*p* < 0.05).

**Results:** In the standing on one-foot state, the peak stress of the porous Ti6Al4V block was 12.42 MPa and that of the UHMWPE block was 19.97 MPa, which is close to its yield stress (21 MPa). Meanwhile, the stress distribution of the UHMWPE block was uneven. In the flexion state, the peak stress of the porous Ti6Al4V block was 16.28 MPa, while that of the UHMWPE block was 14.82 MPa. Compared with the porous Ti6Al4V block group, the average stress of the region of interest in UHMWPE block group was higher in the standing on one foot state and lower in the flexion state (*p* < 0.05).

**Conclusion:** More uniform stress distribution was identified in the porous Ti6Al4V block application which could reserve more bone. On the contrary, uneven stress distribution and a larger high-stress concentration area were found in the UHMWPE block. Hence, the porous Ti6Al4V block is recommended for the treatment of AORI type 3 distal femoral bone defect.

## 1 Introduction

As one of the most effective remedial procedures, total knee revision (TKR) is widely recognized in addressing bone defects following total knee arthroplasty (Theil et al., 2021). According to the Anderson Orthopedic Research Institute (AORI) classification of bone defects (Aggarwal and Baburaj, 2020), AORI type 3 bone defects, which create severe knee dysfunction, often involve femoral condyles and tibial plateau. The management of AORI type 3 bone defects in TKR remains complicated due to a lack of restoration procedures that perfectly fit the sizes of the defects (Sheth et al., 2017). The major therapies for AORI type 3 distal femoral bone defects are now tumor prosthesis UHMWPE block and metal block augmentation (Aggarwal and Baburaj, 2020).

The tumor prosthesis combined with the UHMWPE block has been applied in bone defects treatment for years. The UHMWPE block could reduce the stress shielding because of the low elastic modulus which is similar to the bone (Sun et al., 2015). And the universal design of the UHMWPE block has a predetermined size, which brings a large convenience for clinic applications (Ji et al., 2019). However, the preset size cannot be changed, making it impossible to match bone defects of varying sizes. As a result, osteotomies that exceeded the extent of the bone defect were always performed to satisfy the design requirement (Peng M. J. et al., 2021).

One way to reduce the osteotomy is to combine the stemmed constrained condylar knee (CCK) prosthesis with metal block augmentations (Kornah et al., 2019). Due to the customizability of the metal block which allows the metal block to match the bone defects, more host bone could be reserved (Kang et al., 2019). Additionally, the metal blocks are constructed of Ti6Al4V, which is stiffer than human bone, and so allows for better bone preservation due to its reliable metal structural support (Innocenti and Pianigiani, 2018). Because of the stiffness mismatch between bone and metal, the stiffer metal carries the majority of the stress and the bone just a minor portion. Stress shielding is produced by this imbalanced load assignment (Kang et al., 2019). According to Wolff’s law, the high-stress state on the bone promotes the increase of bone mineral density, while the low stress will decrease bone mineral density and even generate bone resorption (Lipphaus and Witzel, 2018). As a result, proper stress distribution on the bone is important for bone density maintenance and the prevention of bone resorption. Porous metals, which are commonly used in orthopedics, may help to distribute stress more effectively on bones (Faizan et al., 2017; Lei et al., 2021). Studies have shown that the porous Ti6Al4V augmentation has a low equivalent elastic modulus, which could limit stress shielding (Arabnejad et al., 2017; Guoqing et al., 2021). Meanwhile, the osseointegration capacity and biocompatibility of porous Ti6Al4V structure are beneficial for long-term stability (Liu T. et al., 2020; Wang et al., 2020).

Due to the merits and shortcomings of each technique, this study compared porous Ti6Al4V block and UHMWPE block for the treatment of AORI type 3 distal femoral bone defects by finite element analysis (FEA) and provided a reference for the clinical decisions from the standpoint of biomechanics.

## 2 Materials and Methods

### 2.1 Sample Information

The Computed Tomography (CT) images of knee joints used in this study were scanned by Aquilion One scanner (Toshiba, Japan), which was provided by the Orthopedic Research Center of the Second Hospital of Jilin University. The patient is a 57-year-old male who weighs 70 kg and has left knee arthritis. The DICOM data were imported into Mimics Medical V21 (Materialise, Belgium) and the Three Dimension (3D) model of the left femur was reconstructed. This study was approved by the ethics society of the Second Hospital of Jilin University and the informed consent of the patient.

### 2.2 Establishment of Heterogeneous Bone Model and Operation

**FIGURE 1:**
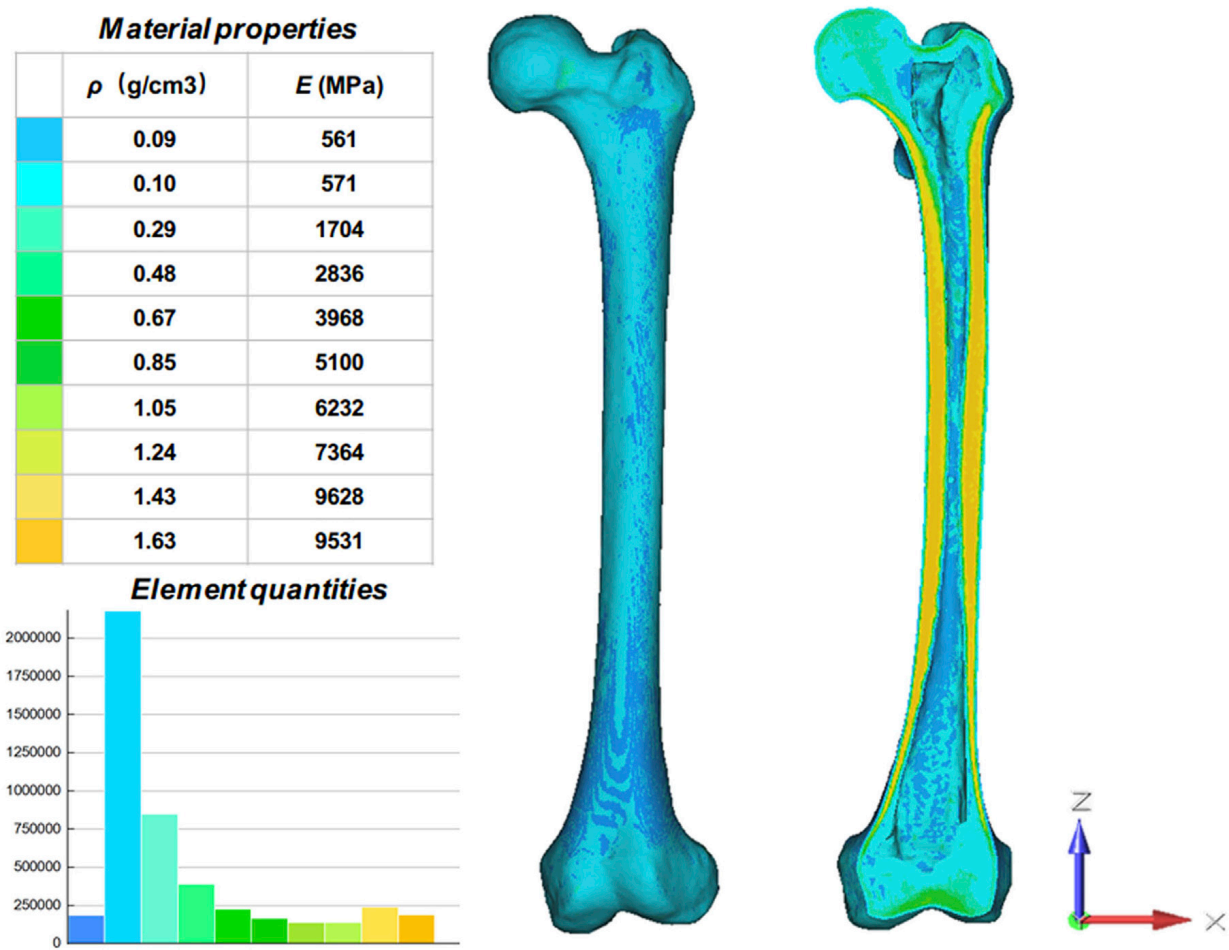
Material properties of the inhomogeneous femur. The femur was divided into ten material properties with ten different colors. ρ: Bone density; E: Elastic modulus.

The knee prosthesis and stem data used in this study were imported into Materialise Magics V21 (Materialise, Belgium) in STL format. Material assignment model with ten distinct Hounsfield Units (HU) zones were obtained by Mimics based on the varied degrees of ray absorption in human bone (Figure 1). According to the formula obtained in previous studies (Liu Y. et al., 2020), the bone density (ρ) and elastic modulus (E) of the femur can be calculated:

$$\rho \left(g/m^{3}\right)=-13.4+1017\times \mathrm{GV}\left(\mathrm{HU}\right)$$

$$E\left(\mathrm{Pa}\right)=-388.8+5925\times \rho \left(g/m^{3}\right)$$

**FIGURE 2:**
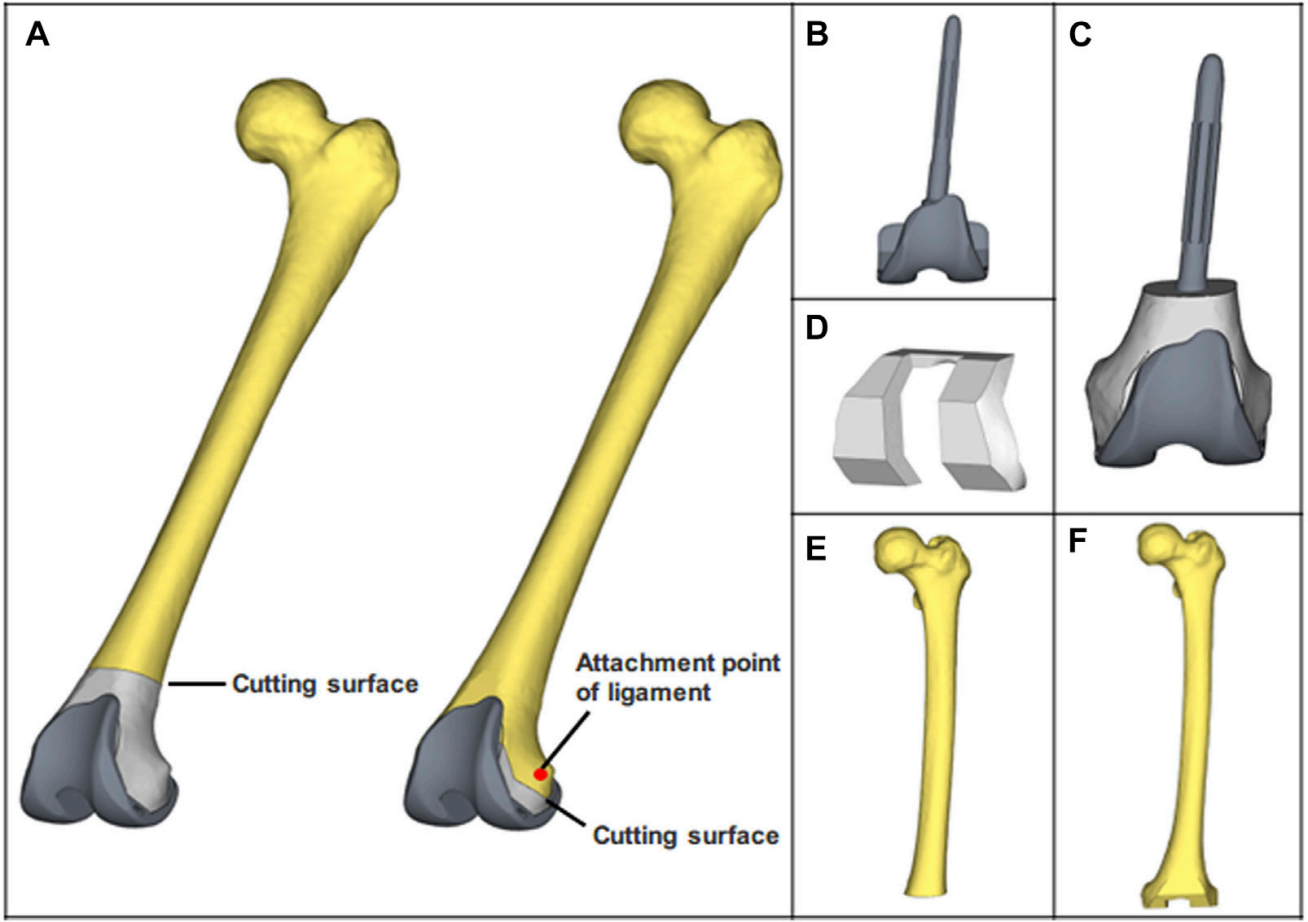
The finite element models of all components. **(A)** Porous Ti6Al4V block and customized prosthesis assembled to simulate the operation. **(B)** CCK prosthesis. **(C)** customized prosthesis which includes a UHMWPE block. **(D)** Porous Ti6Al4V block. **(E, F)** Distal femur osteotomy models, which are paired with porous Ti6Al4V block and UHMWPE block.

Based on the establishment of the heterogeneous femoral model, osteotomy of the distal femur was performed according to the standard surgical requirements in Materialise Magics V21, the cutting operation was performed on the distal 10 mm of the attachment point of the lateral and medial collateral ligament. Boolean subtraction was performed to obtain two distinct surgical approaches (Figure 2A). Through reverse reconstruction, the metal block with customized structural features was created. Similarly, the UHMWPE block was obtained. Rather than constructing the porous structure, the equivalent elastic modulus of the porous Ti6Al4V was used, as the focus of this study was the biomechanical performance of blocks (Burastero et al., 2020). Then, two distinct types of blocks were produced by assigning the properties of UHMWPE and porous Ti6Al4V (Figure 2 C, D). After completing these preparations, all components (prosthesis, bone cement, bone defect filling block, and femur) were assembled precisely. Additionally, the simulated operation meets the requirements for the mechanical and physiological axis alignment following conventional knee arthroplasty.

### 2.3 Sensitivity Analysis

After completing the model reconstruction, sensitivity analysis was performed to reduce the errors caused by different grid densities. Additionally, under the same loading and boundary conditions, the maximum stress on the femur was recorded in five mesh groups (0.5, 0.8, 1.0, 1.2, and 1.5 mm) (table 1). The reference group of unit size 0.5 mm was defined because it more accurately reflects the geometry of the model. The maximum stress difference between the experimental group and the reference group was assessed to be within the range of 5% in order to preserve prediction accuracy (Shriram et al., 2017). The results indicated that case 1 was the optimal decision, as it maintained accuracy while minimizing unnecessary computational resources consumption. As a result, 0.8 mm was the final mesh size of the femur model.

**TABLE 1:** Sensitivity analyses on mesh density for bone.

Case	Element Size (mm)	Number of Elements	Peak Stress in Bone (MPa)
References	0.5	2927914	40.21
Case 1	0.8	1260433	42.20
Case 2	1.0	1433512	35.73
Case 3	1.2	534169	43.00
Case 4	1.5	313700	35.58

## 2.4 Material Parameters and Load Setting

For meshing and material property assignment, all components were imported into Hypermesh V20 (Altair Engineering, United States). To preserve the precise details of the geometry, the mesh size of the prosthesis, cement, and block was 0.5 mm. The material properties of each component were presented in table 2. The equivalent elastic module and Poisson’s ratio of the porous Ti6Al4V was acquired from previous study (Burastero et al., 2020; Song et al., 2021). The contact surface type between different parts was defined as face-to-face contact. In addition, the frozen state, a contact type without movement, was established between bone and bone cement, prosthesis and bone cement, UHMWPE block and bone cement, and porous Ti6Al4V block and bone cement respectively. Additionally, the stick state which allows a small displacement of different contact parts was established between bone and prosthesis, prosthesis and porous Ti6Al4V block, and prosthesis and UHMWPE block (table 3).

**TABLE 2:** Material properties of the components.

Component	Elasticity Modulus (MPa)	Poisson’s Ratio
Prosthesis (Ti6Al4V)	114500	0.3
UHMWPE block	2300	0.3
Porous Ti6Al4V block	25000	0.35
Cement	2150	0.3

**TABLE 3:** Contact types between components.

Contact Surface a	Contact Surface B	Contact Type
Bone	Cement	Freeze
Prosthesis	Cement	Freeze
UHMWPE block	Cement	Freeze
Porous Ti6Al4V block	Cement	Freeze
Prosthesis	Bone	Stick
Prosthesis	UHMWPE block	Stick
Prosthesis	Porous Ti6Al4V block	Stick
Bone	UHMWPE block	Stick
Porous Ti6Al4V block	Bone	Stick

**FIGURE 3:**
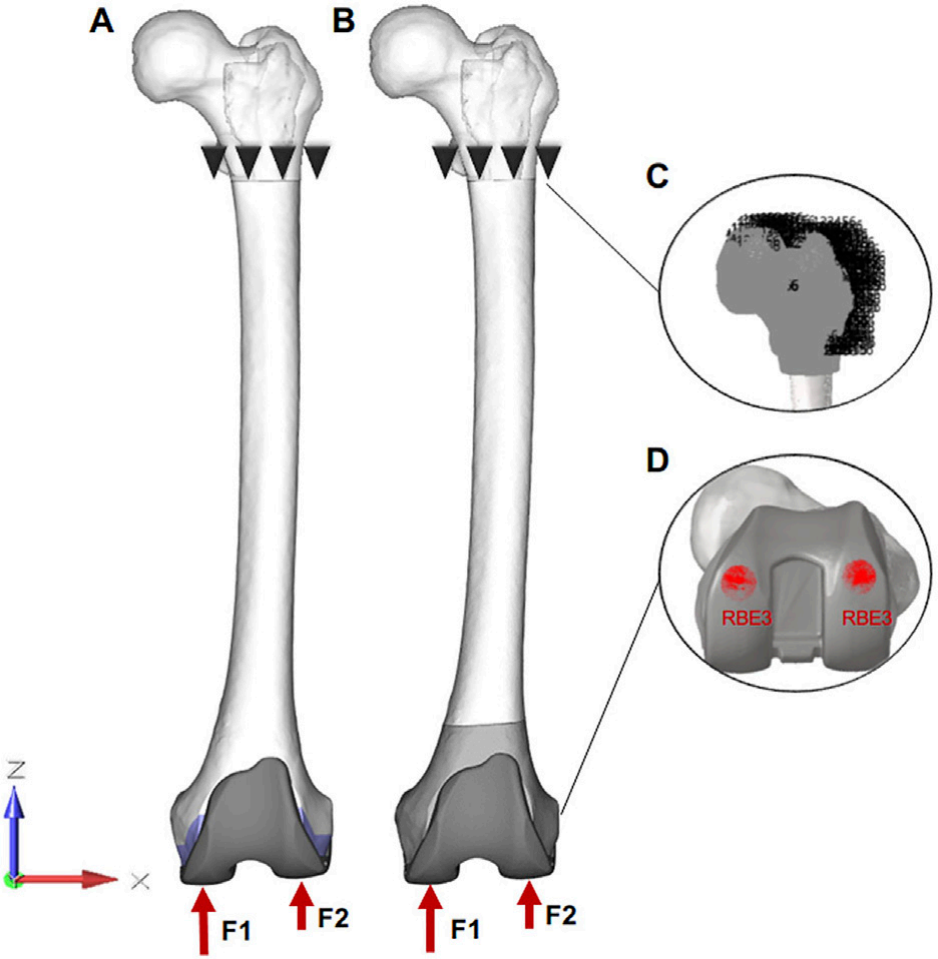
The final finite element model of surgical procedures. **(A, B)**: Loads and constraint conditions on the femur. F1 and F2 represent the force on the medial and lateral femur, respectively. **(C)** all points of the proximal femur are limited to 0 in six degrees of freedom. **(D)** forces are applied to the “Rigid body element three” area in Hypermesh, which can make the force more uniform.

To make this study more realistically, the biomechanical simulations of different motions were carried out: 1. Standing on one foot: the knee joint is in the extension position and the load is up to 2.5 times body weight (BW) (Tang et al., 2017; Innocenti and Pianigiani, 2018); 2. Gait (30° of flexion): the femur is flexion 15° relative to the longitudinal axis of the human body and the load is about 3 times BW (Burastero et al., 2020). Based on the results of previous studies, the load ratio of the medial and lateral condyle of the femur is 60%: 40% (Innocenti and Pianigiani, 2018). Therefore, the loading assignment of the medial and lateral condyle were 1050 N and 700 N in the standing on one-foot state. Similarly, under the condition of knee flexion, the loads of the medial and lateral condyle were 1320 N and 880 N. Meanwhile, the Rigid body element three in Hypermesh was set for more even forces on the surfaces (Zhang et al., 2020). Because the main object of this study is the distal femur, the proximal femur was fixed under the lesser trochanter and all points of the proximal femur were limited to 0 in six degrees of freedom (Figure 3).

### 2.5 Finite Element Simulation

**FIGURE 4:**
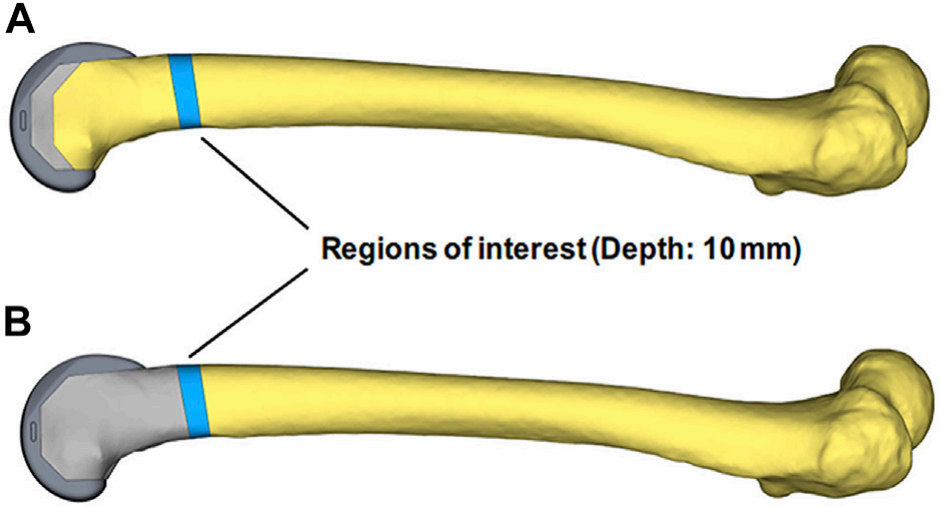
The regions of interest (ROI) definition of the two groups’ models. The blue areas of **(A, B)** were the selected regions of interest, located in the same 10 mm femoral scale above the UHMWPE block.

The FEA was performed by Hypermesh and the post-processing of the results was carried out by Hyperview (Altair Engineering, United States). To more precisely analyze stress transfer between blocks and femurs, the regions of interest (ROI) were designated as the 10 mm scales of the femur above the UHMWPE block and the same femoral portion of the porous Ti6Al4V block group (Innocenti and Pianigiani, 2018). The average Von Mises stresses of ROIs under different motions were analyzed in SPSS V21 (IBM, United States) and the independent samples *t*-test was performed, the *p*-value less than 0.05 was considered to have a significant difference (Figure 4).

## 3 Results

### 3.1 Von Mises Stress Distribution of Blocks in Different Groups

**FIGURE 5:**
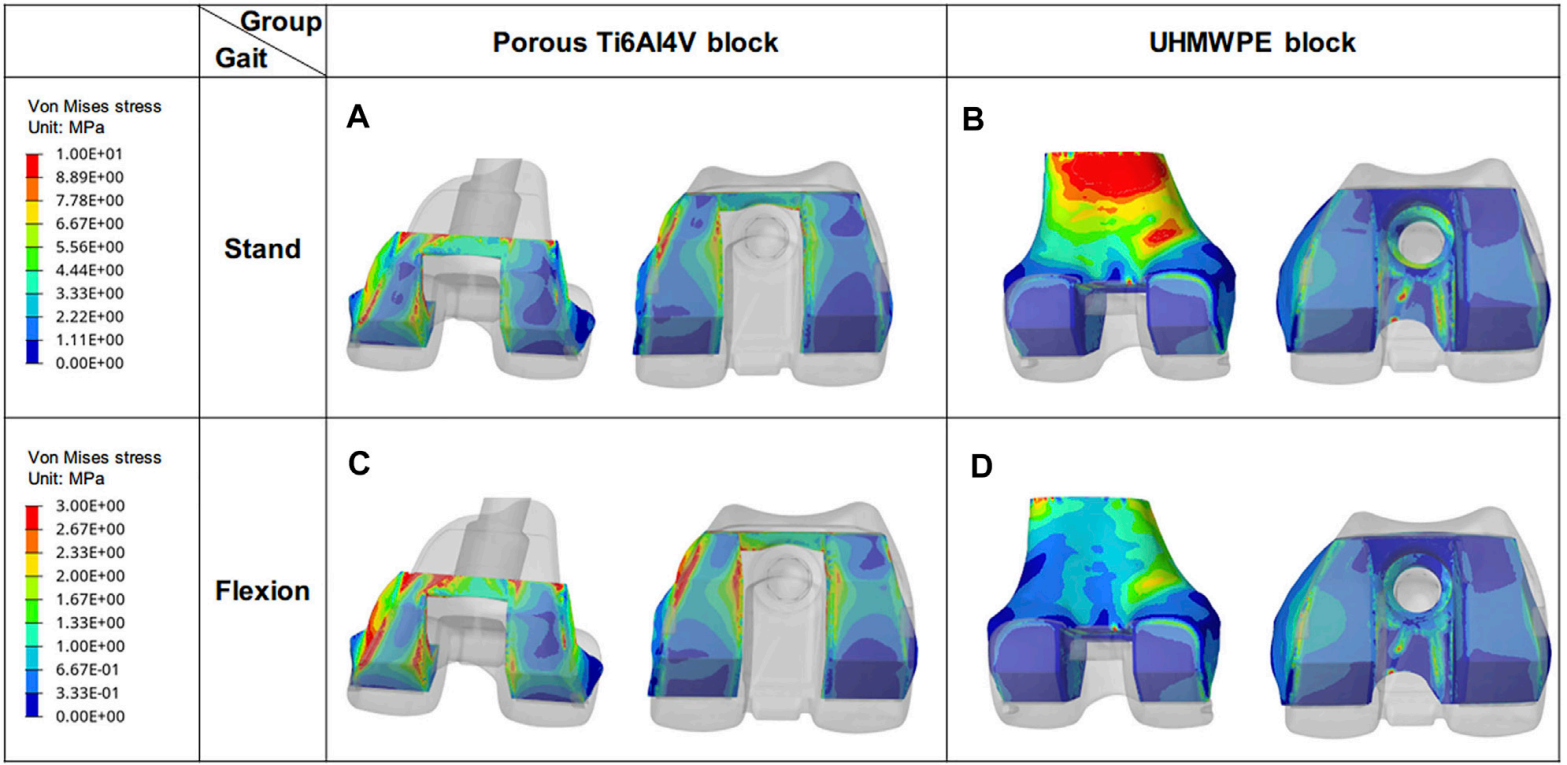
Von Mises stress distribution of blocks under different conditions. **(A)** and **(B)** are the stress distributions of porous Ti6Al4V Block and UHMWPE Block in the standing state, respectively. **(C)** and **(D)** are their stress distribution in the flexing state.

In standing on one-foot state, the maximum stress of the porous Ti6Al4V block and UHMWPE block was 12.42 MPa and 19.97 MPa, respectively (Figure 5). And a large stress concentration was observed on the posterior medial side of the UHMWPE block. At the same time, it was found that the maximum stress appeared in the area in contact with the femur, and the maximum stress (19.97 MPa) of the UHMWPE block was very close to the yield stress (21 MPa) of UHMWPE (Sun et al., 2015). In the knee flexion, the maximum stress of the porous Ti6Al4V block and UHMWPE block was 16.28 and 14.82 MPa respectively. And the maximum stress of the UHMWPE block decreases by 25.79% compared to standing on a one-foot state.

### 3.2 Von Mises Stress Distribution of Femur

**FIGURE 6:**
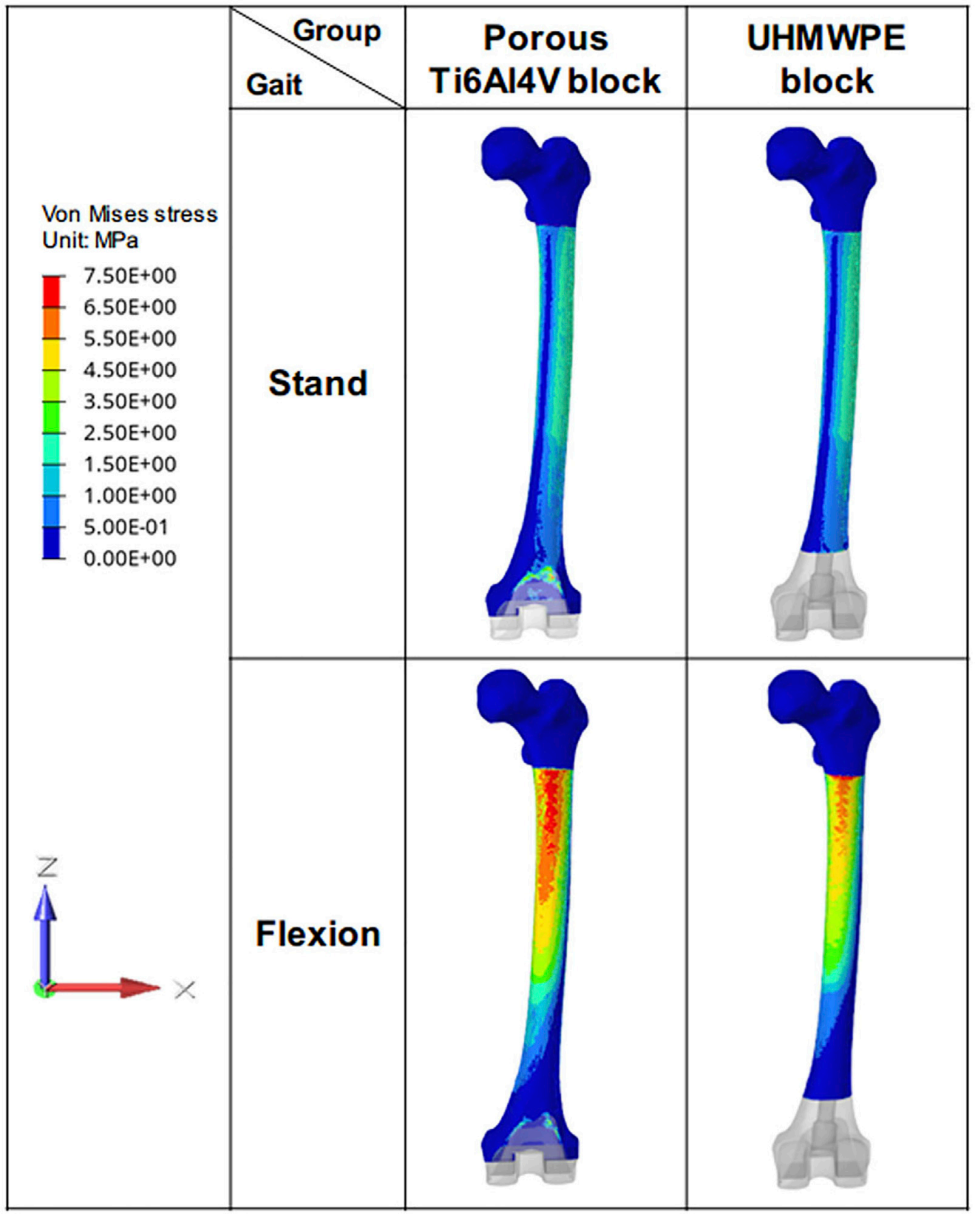
Von Mises stress distribution of femur. The figure shows the stress distribution of femurs in different groups during the standing and flexion stages.

For all the investigated cases, the Von Mises stress decreased gradually from the proximal femur to the distal femur (Figure 6). In the standing on one-foot state, the maximum stress of the femur in porous Ti6Al4V block and UHMWPE block was 36.58 MPA and 35.97 MPa. In the knee flexion state, the maximum stress of the femur in porous Ti6Al4V block and UHMWPE block was 125.1 MPa and 90.55 MPa. The bone stress of the porous Ti6Al4V block group increased significantly, which was 27.62% higher than that of the UHMWPE Block group. Meanwhile, the high-stress distributing area on the bone was larger, indicating that more stress was transferred to the bone.

### 3.3 Von Mises Stress Distribution of Prosthesis

**FIGURE 7:**
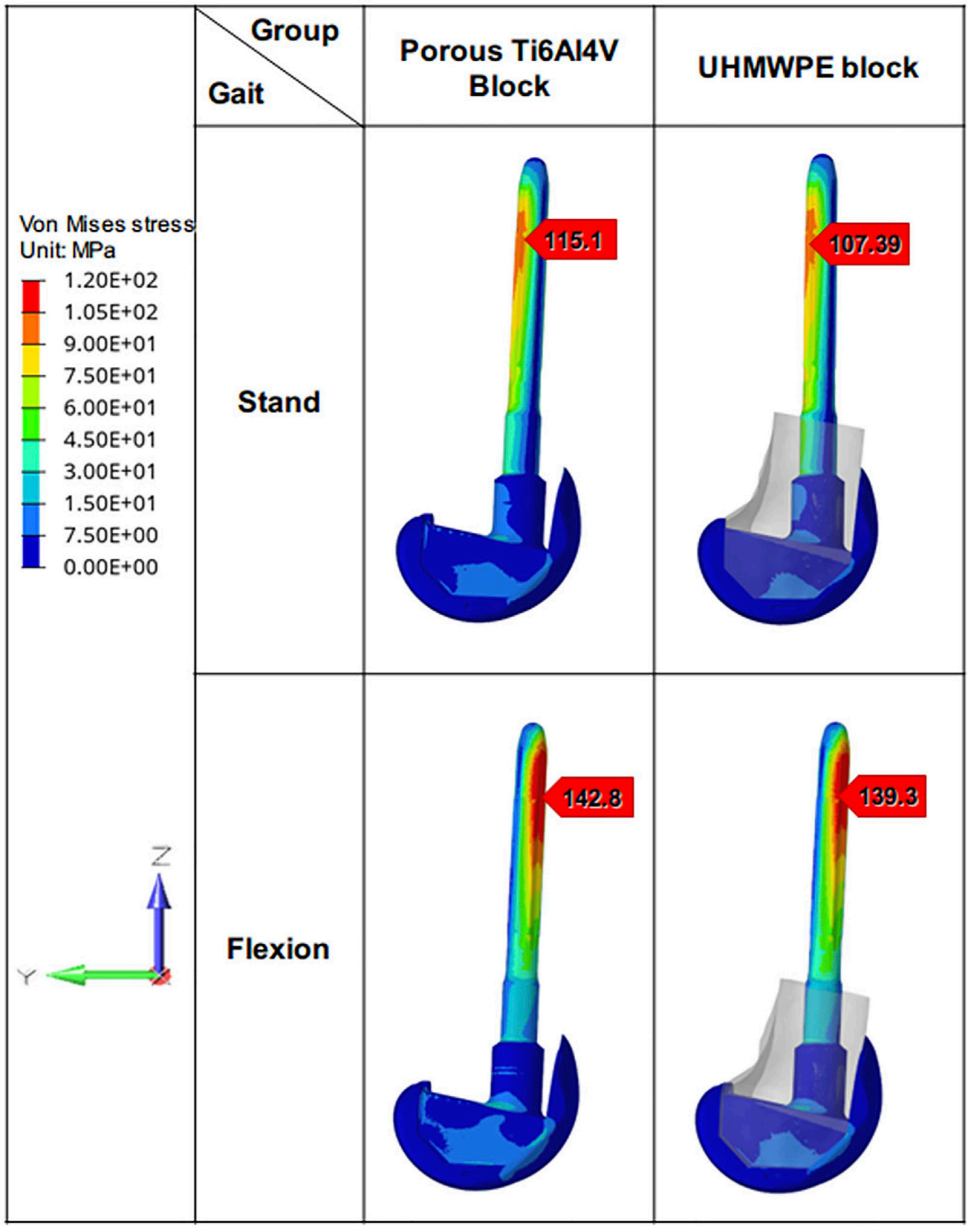
Von Mises stress distribution of different prostheses. The Von Mises stress distribution on the prostheses and its maximum stress under different loading patterns.

The Von Mises stress distribution on the prostheses in different states was shown in Figure 7. In the standing on one-foot state, the peak stress of prostheses in the porous Ti6Al4V block group and UHMWPE block group was 115.1 MPa and 107.39 MPa, respectively. In the flexion state, the peak stress of prostheses in the porous Ti6Al4V block group and UHMWPE block group was 142.8 MPa and 139.3 MPa. Similar to the stress distribution of the femur, there is little difference in stress distribution between prostheses of different groups under the same condition.

### 3.4 The Average Von Mises Stress of the ROIs

The average stress of the ROIs of the two blocks groups under different motions was shown in Table 4. The results showed that for the ROI of the porous Ti6Al4V block group, the Von Mises stress in the flexion was higher than standing on one-foot state, while for the ROI of the UHMWPE group, the Von Mises stress in the flexion was lower than that in the standing on one-foot state. For the average stress of the ROI in the UHMWPE block, it was higher than that of the porous Ti6Al4V block in the standing on one foot state and lower in the flexion state.

**TABLE 4:** Average Von Mises stress (MPa) of ROI.

Group	Stand ± std (MPa)	Flexion ± std (MPa)
Porous Ti6Al4V block	0.147 ± 0.054	0.330 ± 0.051
UHMWPE block	0.400 ± 0.075	0.225 ± 0.063

## 4 Discussion

The management of bone defects is a challenging issue in TKR (Mozella and Cobra, 2021). Both the porous Ti6Al4V block and the UHMWPE block are suitable for AORI type 3 bone defects treatment (Lei et al., 2019; Aggarwal and Baburaj, 2020). However, there is no consensus on which is superior due to the unique properties of each technique. To address this issue, this study compared the porous Ti6Al4V block and UHMWPE block in treating AORI type 3 bone defect in TKR from the view of biomechanics.

FEA is an effective tool to analyze biomechanical performance. The accuracy of the analysis depends on the authenticity of the models and the validity of the loading conditions. The current study reconstructed a heterogeneous femoral model which could realistically reflect the biomechanical performance of diverse bone densities (Saeidi et al., 2020; Ghaziani et al., 2021). Meanwhile, the accuracy of the heterogeneous model reconstruction method in this study has been demonstrated by previous biomechanical tests (Zhang et al., 2020). In this study, the static analysis of standing on one-foot and 30° flexion of the knee was conducted, and both conditions replicated the maximum axial force during gait (Innocenti and Pianigiani, 2018; Burastero et al., 2020). Additionally, these conditions are the crucial training contents of postoperative rehabilitation (Chen et al., 2021). Therefore, the evaluation of these two conditions is beneficial for analyzing the biomechanical performance of the knee joint system. The application of the heterogeneous bone model and complex load conditions guaranteed the credibility and accuracy of the experimental data.

Several evaluation indexes were included for the fairness of the result evaluation. The peak stress, the distribution of stress, and the average stress of ROI were mainly studied from the biomechanical perspective.

The peak stress was evaluated in this study to assess the biomechanical performance and the stress transfer capacity of different blocks. The maximum stress (19.97 MPa) of the UHMWPE block was close to its yield stress (21 MPa) (Sun et al., 2015). And the peak stress of blocks which is close to the yield stress is regarded as a risk factor for deformation (Arab et al., 2020). Therefore, attention must be paid to this fact which might shorten the service life of the UHMWPE block. Because the loads of the knee-prosthesis-metal block system increased when the loads altered from the standing on one-foot state to the flexion state, the stress of the porous Ti6Al4V block increased from 12.42 MPa to 16.28 MPa, while the stress of the UHMWPE block decreased relatively due to its lower elastic modulus than prosthesis and human bone. In addition, combined with the stress distribution of prosthesis, it was found that when the loads application was altered to the flexion state, the maximum stress of the prosthesis in UHMWPE block group increased more (30%) than that of the porous Ti6Al4V block group (24%), which indicated that more stress was transferred to the prosthesis of the UHMWPE group under the flexion state, suggesting more stress shielding occurred in the UHMWPE block group. Additionally, the peak stress of the femur is considered to be an important index to measure the stress transfer ability of blocks (Burastero et al., 2020). The maximum stress in the porous Ti6Al4V block group was 1.67% higher in the standing on one-foot state and 27.62% higher in the flexion state compared to the UHMWPE block group. The results showed that more stress was borne by the bone in the porous Ti6Al4V block group, indicating a better stress transfer capacity.

Another key indication for assessing stress transfer is the stress distribution. The uneven high-stress concentration of the block represents inadequate stress transfer capability and uneven mechanical stimulation (Peng M. J. et al., 2021; Vogel et al., 2021). In this study, a larger high-stress concentration area was found on the UHMWPE block which has been proved to be a challenge for long-term use (Arab et al., 2020). On the contrary, more uniform stress distribution and less high-stress concentration area were identified on the porous Ti6Al4V block (Figure 5), indicating a better stress transfer capability (Liu T. et al., 2020). At the same time, a broader and more uniform area of high-stress distribution was observed on the anterior and posterior sides of the bone in the porous Ti6Al4V block group. The existence of these even mechanical stimulations is advantageous for bone growth in the porous structure (Peng W.-m. et al., 2021; Song et al., 2021).

In this study, the average stress of the ROIs in the two groups were measured for the evaluation of the stress transfer capacity. Innocenti et al. have demonstrated that the ROI which is the 10 mm area close to the contacting surface of the metal blocks are sensitive for the stress changes. (Innocenti and Pianigiani, 2018). And in the present paper, the ROIs of these two techniques were set at the same 10 mm femoral areas for the evaluation of the stress transfer capacity. The statistical study of the average stress in the ROI of the UHMWPE block and the porous Ti6Al4V block revealed significant differences (*p* < 0.05). It’s worthwhile to notice that under the flexion state, the average Von Mises stress of ROI in the UHMWPE group was lower than that of the standing on one-foot state, which was related to stress shielding between the prosthesis and UHMWPE block. For the standing on one foot state, the result of the UHMWPE block’s ROI was higher than that of the porous Ti6Al4V block (table 4). The explanation for the discrepancy is that the elastic modulus of UHMWPE is relatively lower than that of the porous Ti6Al4V (Bagudanch et al., 2018).

The above findings indicated that the application of porous Ti6Al4V block could provide superior biomechanical performance while maintaining the reliable structural strength under the investigated situations. At first, from the viewpoint of structural function, the use of porous Ti6Al4V block greatly decreases unnecessary osteotomy, ensuring the integrity of the knee joint and the rehabilitation of knee joint activity after the operation (Oussedik et al., 2020). On the other hand, the porous Ti6Al4V block reduced the stress shielding in terms of stress transfer and its mechanical property was better than that of the UHMWPE block. Furthermore, it is worth mentioning that the existence of porous structures promotes the osseointegration, which has been demonstrated to improve the long-term stability (Winther et al., 2016; Lei et al., 2021). Therefore, these findings provide a new possibility for the treatment of TKR bone defects and pave the way for the design and optimization of metal block augmentations.

This study provided a promising option for clinics, nonetheless, some limitations of this study should also be emphasized. First of all, although the sensitivity of the femoral model has been analyzed and the best mesh size was selected, there is still a lack of comparative verification in the mechanical testing, which will be completed in the following stage. Second, the statics analysis of specified situations was performed in this study and the credibility of the statics analysis has been demonstrated previously (Zhang et al., 2020; Shu et al., 2021), whereas it’s difficult to evaluate the biomechanical performance of the gait cycle entirely. Therefore, the dynamic analysis will be incorporated in the future study.

## 5 Conclusion

In this study, the biomechanical performance of porous Ti6Al4V block and UHMWPE block in treating AORI type 3 distal femoral bone defect of TKR was evaluated by FEA. The results revealed that the porous Ti6Al4V block could reduce stress shielding, reserve more bone, and provide stable structure support, implying that the porous Ti6Al4V block might have superior biomechanical performance to the UHMWPE block. These findings could provide a biomechanical reference for the selection of treatment methods in clinics.

## Data Availability

The original contributions presented in the study are included in the article/Supplementary Material, further inquiries can be directed to the corresponding authors.

## References

[B1] Aggarwal A. K. Baburaj V. (2020). Managing Bone Defects in Primary Total Knee Arthroplasty: Options and Current Trends. Musculoskelet. Surg. 105, 31–38. 10.1007/s12306-020-00683-7 33058073

[B2] Arab A. Z. E.-A. Merdji A. Benaissa A. Roy S. Bachir Bouiadjra B.-A. Layadi K. (2020). Finite-Element Analysis of a Lateral Femoro-Tibial Impact on the Total Knee Arthroplasty. Comput. Methods Programs Biomed. 192, 105446. 10.1016/j.cmpb.2020.105446 32200048

[B3] Arabnejad S. Johnston B. Tanzer M. Pasini D. (2017). Fully Porous 3D Printed Titanium Femoral Stem to Reduce Stress-Shielding Following Total Hip Arthroplasty. J. Orthop. Res. 35, 1774–1783. 10.1002/jor.23445 27664796

[B4] Bagudanch I. García-Romeu M. L. Ferrer I. Ciurana J. (2018). Customized Cranial Implant Manufactured by Incremental Sheet Forming Using a Biocompatible Polymer. Rpj 24, 120–129. 10.1108/rpj-06-2016-0089

[B5] Burastero G. Pianigiani S. Zanvettor C. Cavagnaro L. Chiarlone F. Innocenti B. (2020). Use of Porous Custom-Made Cones for Meta-Diaphyseal Bone Defects Reconstruction in Knee Revision Surgery: a Clinical and Biomechanical Analysis. Arch. Orthop. Trauma Surg. 140, 2041–2055. 10.1007/s00402-020-03670-6 33170352

[B6] Chen X. Li X. Zhu Z. Wang H. Yu Z. Bai X. (2021). Effects of Progressive Resistance Training for Early Postoperative Fast-Track Total Hip or Knee Arthroplasty: A Systematic Review and Meta-Analysis. Asian J. Surg. 44, 1245–1253. 10.1016/j.asjsur.2021.02.007 33715964

[B7] Faizan A. Bhowmik-Stoker M. Alipit V. Kirk A. E. Krebs V. E. Harwin S. F. (2017). Development and Verification of Novel Porous Titanium Metaphyseal Cones for Revision Total Knee Arthroplasty. J. Arthroplasty 32, 1946–1953. 10.1016/j.arth.2017.01.013 28196619

[B8] Ghaziani A. O. Soheilifard R. Kowsar S. (2021). The Effect of Functionally Graded Materials on Bone Remodeling Around Osseointegrated Trans-femoral Prostheses. J. Mech. Behav. Biomed. Mater. 118, 104426. 10.1016/j.jmbbm.2021.104426 33740685

[B9] Guoqing Z. Junxin L. Chengguang Z. Juanjuan X. Xiaoyu Z. Anmin W. (2021). Design Optimization and Manufacturing of Bio-Fixed Tibial Implants Using 3D Printing Technology. J. Mech. Behav. Biomed. Mater. 117, 104415. 10.1016/j.jmbbm.2021.104415 33652236

[B10] Innocenti B. Fekete G. Pianigiani S. (2018). Biomechanical Analysis of Augments in Revision Total Knee Arthroplasty. J. Biomech. Eng. 140. 10.1115/1.4040966 30098138

[B11] Ji T. Yang Y. Li D. s. Tang X. d. Guo W. (2019). Limb Salvage Using Non‐hinged Endoprosthesis and Staged Correction of Leg‐length Discrepancy for Children with Distal Femoral Malignant Tumors. Orthop. Surg. 11, 819–825. 10.1111/os.12525 31489784 PMC6819186

[B12] Kang K. Tien T. Lee M. Lee K.-Y. Kim B. Lim D. (2019). Suitability of Metal Block Augmentation for Large Uncontained Bone Defect in Revision Total Knee Arthroplasty (TKA). Jcm 8, 384. 10.3390/jcm8030384 PMC6462980 30893934

[B13] Kornah B. A. Safwat H. M. Abdel-Hameed S. K. Abdel-AAl M. Abdelaziz M. Abuelesoud M. I. (2019). Managing of Post-traumatic Knee Arthritis by Total Knee Arthroplasty: Case Series of 15 Patients and Literature Review. J. Orthop. Surg. Res. 14, 168. 10.1186/s13018-019-1180-3 31151399 PMC6543569

[B14] Lei H. Yi T. Fan H. Pei X. Wu L. Xing F. (2021). Customized Additive Manufacturing of Porous Ti6Al4V Scaffold with Micro-topological Structures to Regulate Cell Behavior in Bone Tissue Engineering. Mater. Sci. Eng. C 120, 111789. 10.1016/j.msec.2020.111789 33545915

[B15] Lei P.-f. Hu R.-y. Hu Y.-h. (2019). Bone Defects in Revision Total Knee Arthroplasty and Management. Orthop. Surg. 11, 15–24. 10.1111/os.12425 30809942 PMC6430493

[B16] Lipphaus A. Witzel U. (2018). Finite‐Element Syntheses of Callus and Bone Remodeling: Biomechanical Study of Fracture Healing in Long Bones. Anat. Rec. 301, 2112–2121. 10.1002/ar.23893 30290071

[B17] Liu T. Chen Y. Apicella A. Mu Z. Yu T. Huang Y. (2020). Effect of Porous Microstructures on the Biomechanical Characteristics of a Root Analogue Implant: An Animal Study and a Finite Element Analysis. ACS Biomater. Sci. Eng. 6, 6356–6367. 10.1021/acsbiomaterials.0c01096 33449664

[B18] Liu Y. Zhang A. Wang C. Yin W. Wu N. Chen H. (2020). Biomechanical Comparison between Metal Block and Cement-Screw Techniques for the Treatment of Tibial Bone Defects in Total Knee Arthroplasty Based on Finite Element Analysis. Comput. Biol. Med. 125, 104006. 10.1016/j.compbiomed.2020.104006 32971324

[B19] Mozella A. d. P. Cobra H. A. d. A. B. (2021). Falhas ósseas nas revisões de artroplastia total Do joelho. Rev. Bras. Ortop. (Sao Paulo) 56, 138–146. 10.1055/s-0040-1713392 33935308 PMC8075647

[B20] Oussedik S. Abdel M. P. Victor J. Pagnano M. W. Haddad F. S. (2020). Alignment in Total Knee Arthroplasty. Bone & Jt. J. 102-B, 276–279. 10.1302/0301-620X.102B3.BJJ-2019-1729 32114811

[B21] Peng M. J. Cao X. Chen H.-Y. Hu Y. Li X. Lao Y. (2021). Intralesional Curettage versus Prosthetic Replacement for Bone Tumors - a Finite Element Analysis Case of Limb Salvage Simulation in Biomechanics. Comput. Methods Programs Biomed. 198, 105775. 10.1016/j.cmpb.2020.105775 33130494

[B22] Peng W.-m. Cheng K.-j. Liu Y.-f. Nizza M. Baur D. A. Jiang X.-f. (2021). Biomechanical and Mechanostat Analysis of a Titanium Layered Porous Implant for Mandibular Reconstruction: The Effect of the Topology Optimization Design. Mater. Sci. Eng. C 124, 112056. 10.1016/j.msec.2021.112056 33947550

[B23] Saeidi M. Gubaua J. E. Kelly P. Kazemi M. Besier T. Dicati G. W. O. (2020). The Influence of an Extra-articular Implant on Bone Remodelling of the Knee Joint. Biomech. Model Mechanobiol. 19, 37–46. 10.1007/s10237-019-01193-7 31300999

[B24] Sheth N. P. Bonadio M. B. Demange M. K. (2017). Bone Loss in Revision Total Knee Arthroplasty. J. Am. Acad. Orthop. Surg. 25, 348–357. 10.5435/JAAOS-D-15-00660 28406878

[B25] Shriram D. Praveen Kumar G. Cui F. Lee Y. H. D. Subburaj K. (2017). Evaluating the Effects of Material Properties of Artificial Meniscal Implant in the Human Knee Joint Using Finite Element Analysis. Sci. Rep. 7, 11. 10.1038/s41598-017-06271-3 28729605 PMC5519683

[B26] Shu L. Sato T. Hua X. Sugita N. (2021). Comparison of Kinematics and Contact Mechanics in Normal Knee and Total Knee Replacements: A Computational Investigation. Ann. Biomed. Eng. 49, 2491–2502. 10.1007/s10439-021-02812-0 34142278

[B27] Song K. Wang Z. Lan J. Ma S. (2021). Porous Structure Design and Mechanical Behavior Analysis Based on TPMS for Customized Root Analogue Implant. J. Mech. Behav. Biomed. Mater. 115, 104222. 10.1016/j.jmbbm.2020.104222 33310682

[B28] Sun C. Wang L. Wang Z. Geng L. Li D. Sui M. (2015). Finite Element Analysis of a Retrieved Custom-Made Knee Prosthesis. J. Mech. Med. Biol. 15, 1550020. 10.1142/s0219519415500207

[B29] Tang F. Zhou Y. Zhang W. Min L. Shi R. Luo Y. (2017). All-polyethylene Tibial Components in Distal Femur Limb-Salvage Surgery: a Finite Element Analysis Based on Promising Clinical Outcomes. J. Orthop. Surg. Res. 12, 57. 10.1186/s13018-017-0555-6 28376828 PMC5381042

[B30] Theil C. Schneider K. N. Gosheger G. Schmidt-Braekling T. Ackmann T. Dieckmann R. (2021). Revision TKA with a Distal Femoral Replacement Is at High Risk of Reinfection after Two-Stage Exchange for Periprosthetic Knee Joint Infection. Knee Surg. Sports Traumatol. Arthrosc. 30, 899–906. 10.1007/s00167-021-06474-2 33564916 PMC8901466

[B31] Vogel D. Wehmeyer M. Kebbach M. Heyer H. Bader R. (2021). Stress and Strain Distribution in Femoral Heads for Hip Resurfacing Arthroplasty with Different Materials: A Finite Element Analysis. J. Mech. Behav. Biomed. Mater. 113, 104115. 10.1016/j.jmbbm.2020.104115 33189013

[B32] Wang C. Xie Q. Yang L. Liu J. Liu D. Li Z. (2020). A 3D Printed Porous Titanium Alloy Rod with Biogenic Lamellar Configuration for Treatment of the Early-Stage Femoral Head Osteonecrosis in Sheep. J. Mech. Behav. Biomed. Mater. 106, 103738. 10.1016/j.jmbbm.2020.103738 32250947

[B33] Winther N. Jensen C. Petersen M. Lind T. Schrøder H. Petersen M. (2016). Changes in bone mineral density of the proximal tibia after uncemented total knee arthroplasty. A prospective randomized study. Int. Orthop. (SICOT) 40, 285–294. 10.1007/s00264-015-2852-1 26183139

[B34] Zhang A. Chen H. Liu Y. Wu N. Chen B. Zhao X. (2020). Customized reconstructive prosthesis design based on topological optimization to treat severe proximal tibia defect. Bio-des. Manuf. 4, 87–99. 10.1007/s42242-020-00102-7

