# Supplementary figures and images for: Biomechanical Comparison Between Porous Ti6Al4V Block and Tumor Prosthesis UHMWPE Block for the Treatment of Distal Femur Bone Defects
Source: Front Bioeng Biotechnol. 2022 Jul 5;10:939371. doi: 10.3389/fbioe.2022.939371 (PMC9294404; doi:10.3389/fbioe.2022.939371)

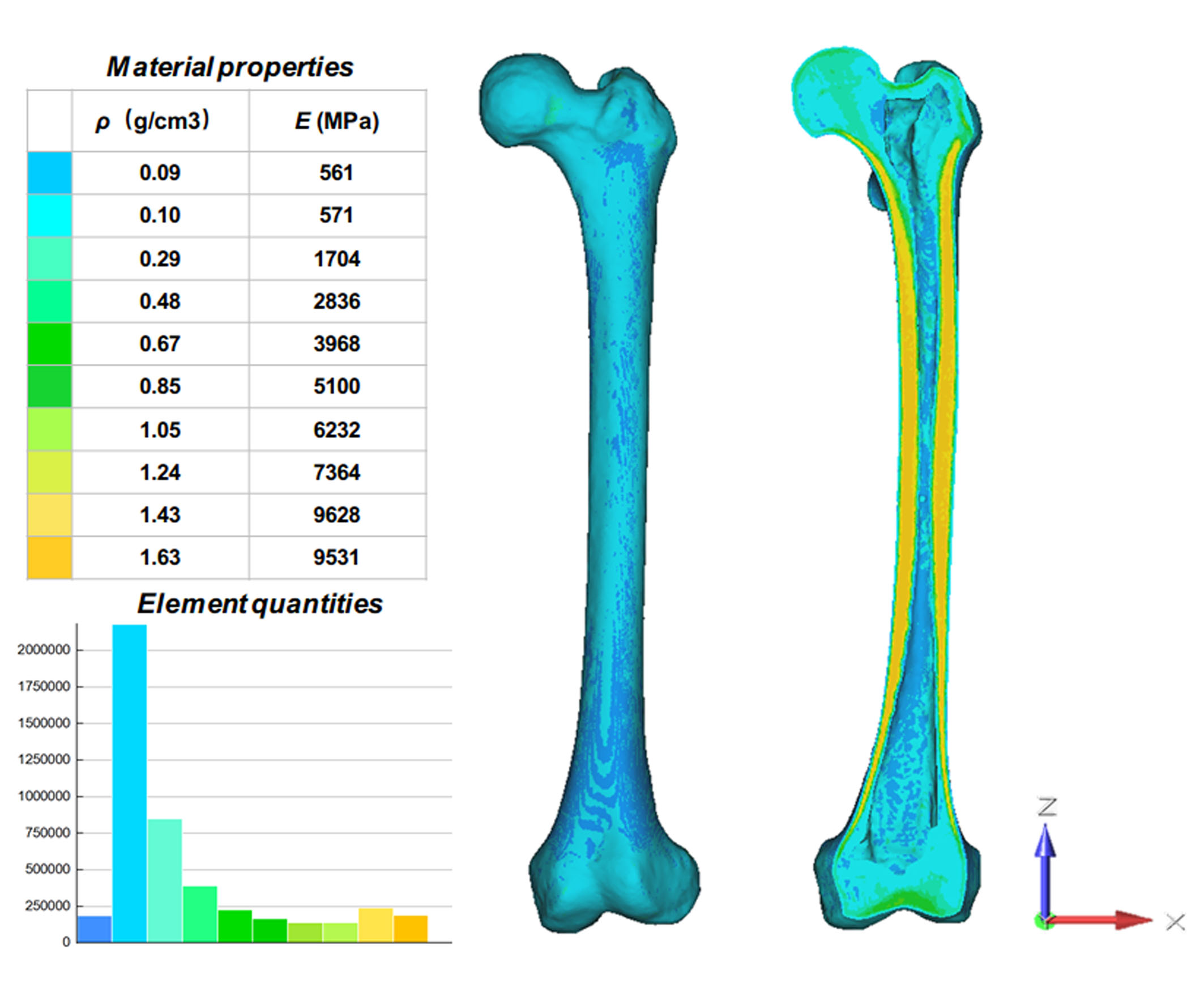

Supplement: Supplementary file 1 [file DataSheet1.ZIP › Fig 1.jpg]

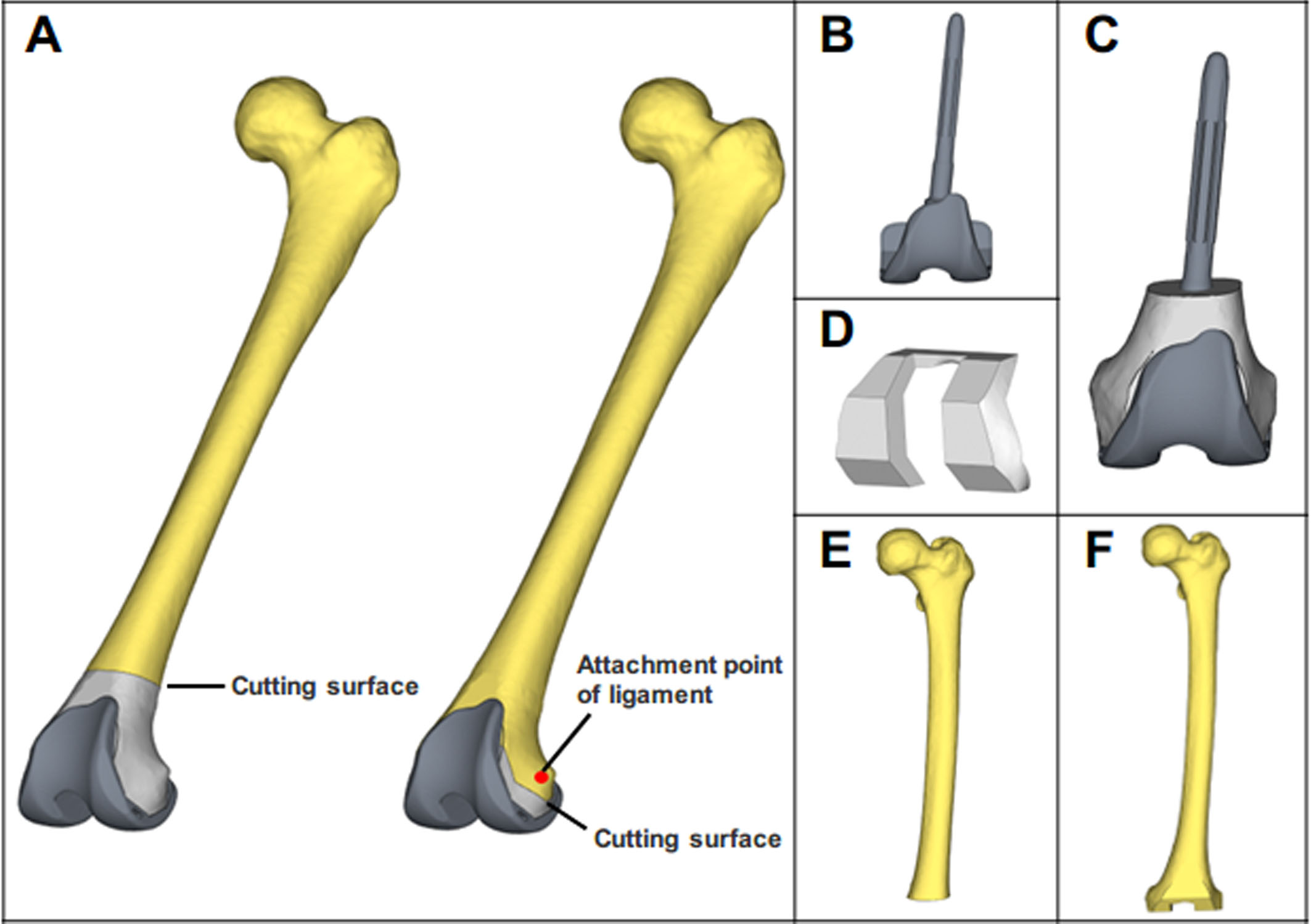

Supplement: Supplementary file 1 [file DataSheet1.ZIP › Fig 2.jpg]

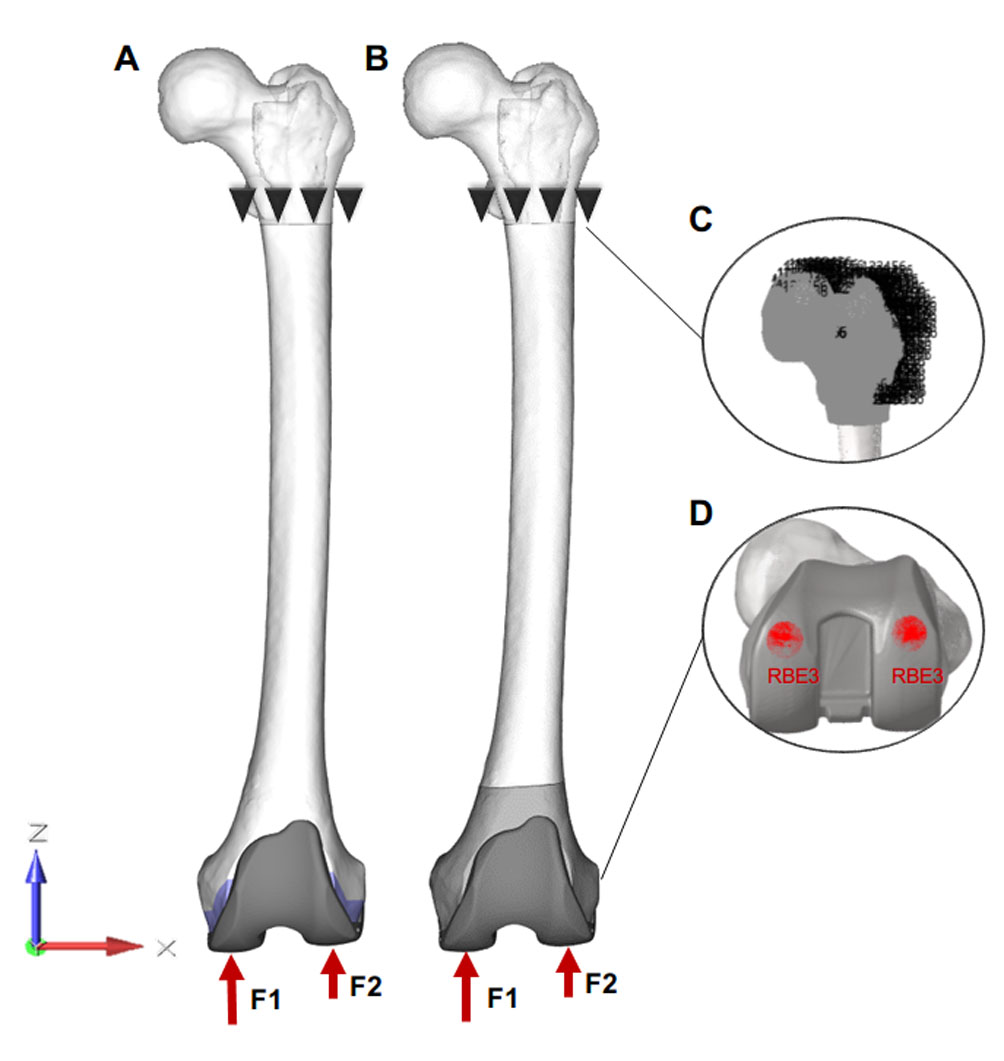

Supplement: Supplementary file 1 [file DataSheet1.ZIP › Fig 3.jpg]

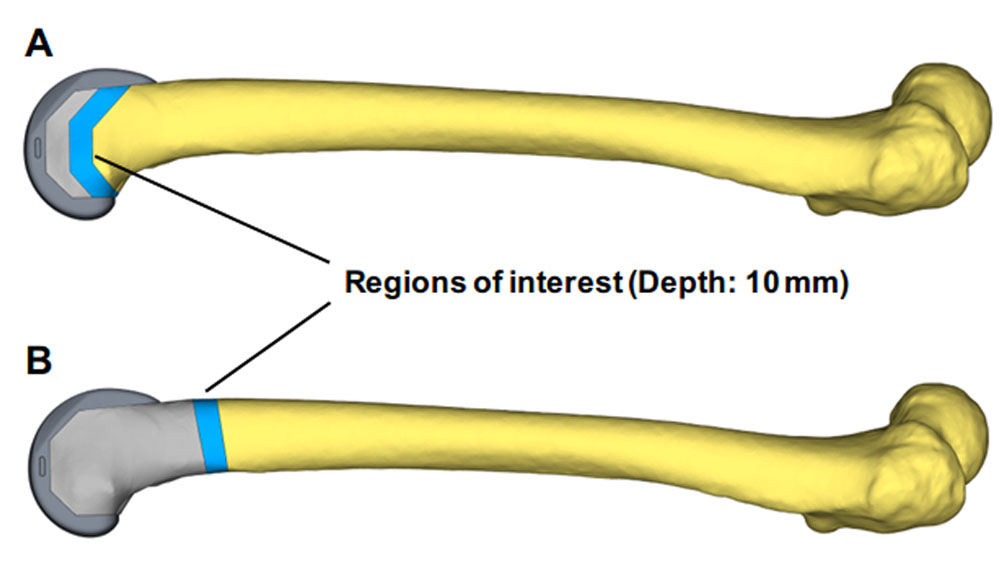

Supplement: Supplementary file 1 [file DataSheet1.ZIP › Fig 4.jpg]

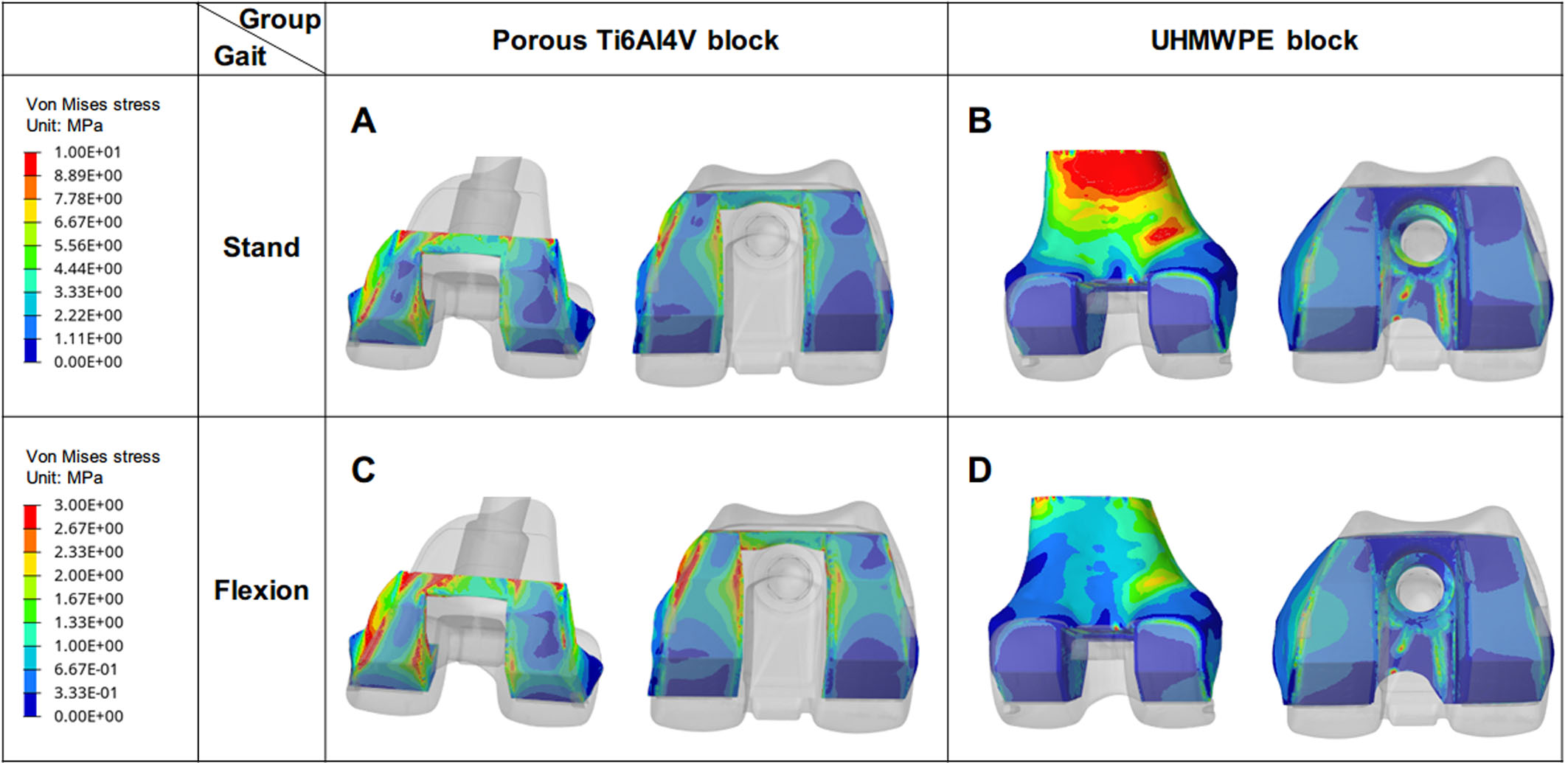

Supplement: Supplementary file 1 [file DataSheet1.ZIP › Fig 5.jpg]

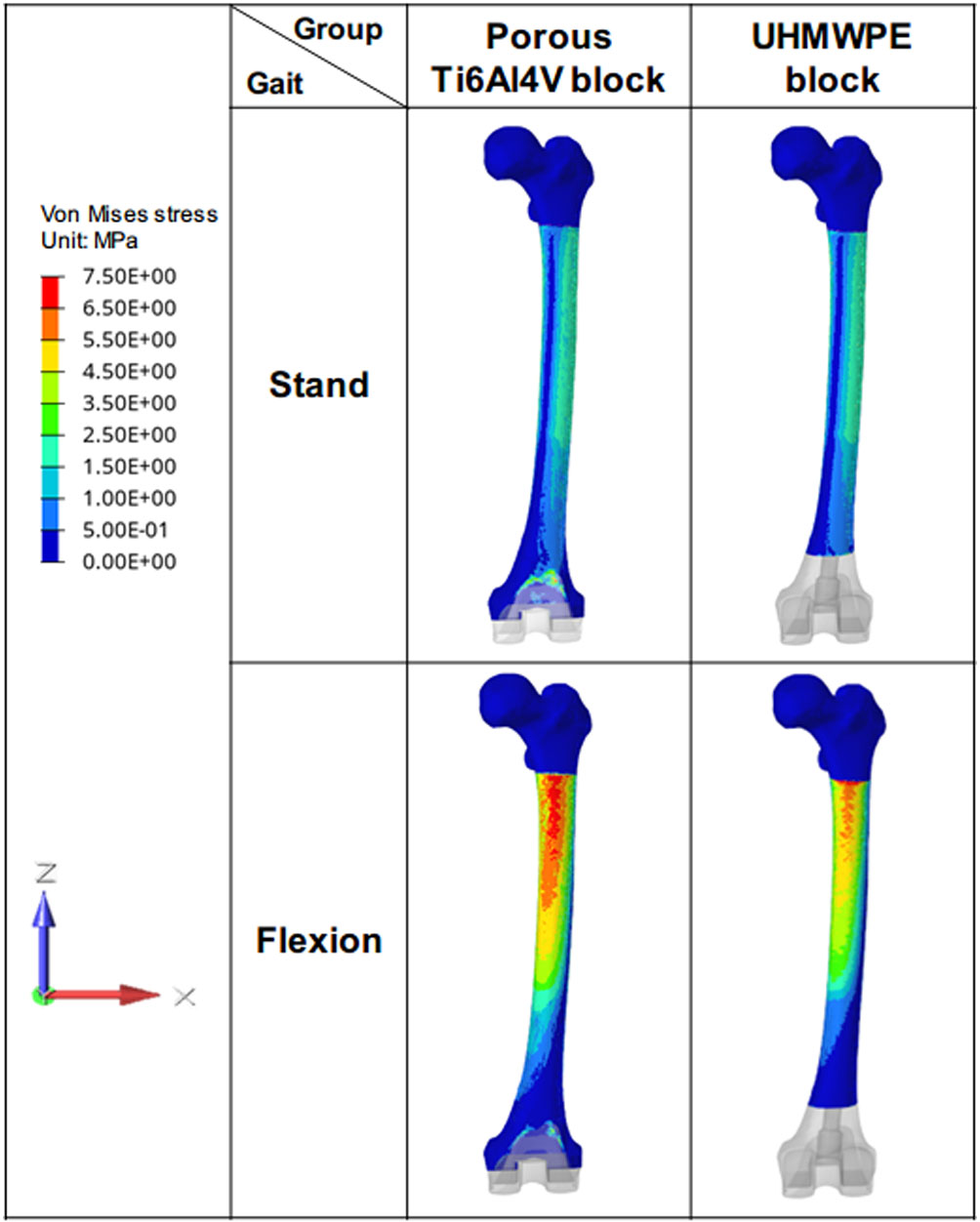

Supplement: Supplementary file 1 [file DataSheet1.ZIP › Fig 6.jpg]

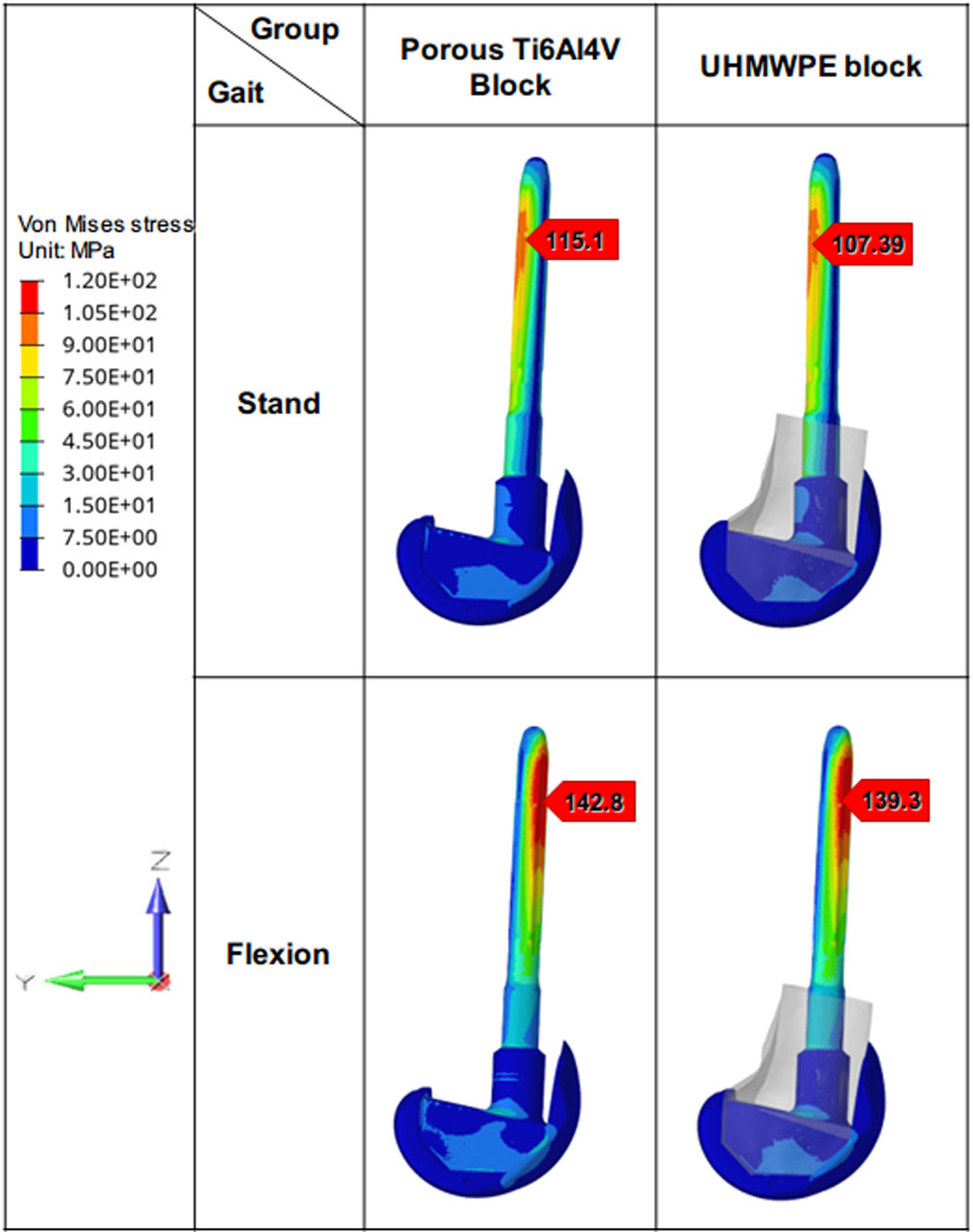

Supplement: Supplementary file 1 [file DataSheet1.ZIP › Fig 7.jpg]
